# Design and Analysis of Electronic Head Protector for Taekwondo Sports

**DOI:** 10.3390/s22041415

**Published:** 2022-02-12

**Authors:** Fayez Qureshi, Sri Krishnan

**Affiliations:** Department of Electrical, Computer, and Biomedical Engineering, Ryerson University, Toronto, ON M5B 2K3, Canada; krishnan@ryerson.ca

**Keywords:** signal analysis, classification algorithm, IMU, point scoring system, taekwondo

## Abstract

Electronic point scoring systems (PSS) for vests are heavily relied upon in taekwondo. However, no classification and assessment of legal and illegal taekwondo techniques exist. This is also referred to as hit-validation and the objective of this research is to create an electronic helmet (eHelmet) for hit-validation. Three main studies were performed to achieve this objective: Robustness Testing, Sensor Placement and Classification of Impacts to the head. The first two studies are preliminary to the main Classification of Impacts study. This is needed as no data sets using an IMU are currently available for taekwondo. Robustness Testing: proved that IMU can in-fact be used in the inherently harsh environments of taekwondo with a linear response. The calculated response for the IMU is: f(x) = mx + b, where m is 0.2947 and b is 1.499 (accelerometer) and f(x) = mx + b, where m is 28.33 and b is 84.8 (gyroscope). Sensor Placement: Qualitatively and quantitatively concluded the ideal location for the sensor and electronics is indeed the back of the head, based on durability, cost, human factors, and signal quality. Classification of Impacts: IMU classified real-world impacts with 90% accuracy. The two classes were roundhouse kick (legal) and punch (illegal). An eHelmet using an IMU is capable of classifying impacts with high accuracy. The benefit of our system includes low cost, lightweight algorithm for on-device computing (edge computing), and real-time classification. Furthermore, it possesses all the safety requirements of current protective headgear.

## 1. Introduction

Taekwondo was officially inducted as an official Olympic Sport in 2000 in the Sydney Games [1,2]. Today, there are 206 national federations that participate in the sport with over 80 million athletes [3]. Countries across the globe compete in the sport, including para-athletes, demonstrating its popularity [3]. World Taekwondo (WT) is the international governing body and was established in 1973. Taekwondo is combative and requires protective head gear that is worn by athletes during competition.

When the sport was introduced to the Olympics, it was a referee-exclusive sport which led to increased controversy surrounding referee/judges’ accuracy, bias and fairness [1,2,4,5,6]. These issues were significant as spectatorship of the sport suffered greatly due to factors such as a short event history, failure of event promotions and marketing and/or ineffective event operations [6]. This combined with the fact that ticket sales represent the primary stream of revenue, became a serious complication [7]. The WT introduced point scoring systems (PSS) to combat this in the 2012 London Olympics with an electronic chest protector. In the 2016 Rio Olympics, the first electronic head protector was introduced [8,9]. As of 2019, there are only two companies that can produce electronic PSS, namely: Daedo and KP&P [3]. Currently, all hits to the head are scored by judges and impacts to the head are worth the most points. For example, a turning kick to the head is worth five points and a normal kick to the head is worth three points. This comprises a significant portion of points currently being rewarded by judges.

As the WT themselves stated “The PSS is in constant process of technical evolution” and hence both these systems have limitations [10]. These limitations include: hit validation and high prices [10,11]. Hit validation refers to the classification and assessment of legal and illegal taekwondo techniques and automatic penalization of illegal strikes. KP&P and Daedo systems have been shown to incorrectly award points on an illegal hit which can lead to false conclusions in high-stake matches. Currently, there is no hit validation on head gear and all points are still scored by judges highlighting this as an under-researched area [10,12]. The next issue regards cost and complexity, current systems cost upwards of USD 2800, requiring several components such as receivers, transmitters, software, judging triggers and scoring boxes [11,13]. To reduce complexity, sensors bearing a consensus for optimal sensor location needs to be investigated [12].

These limitations need to be resolved due to the following points: (1) spectatorship requires transparency and fairness since the rules themselves require headgear to be accurate (2) point scoring systems have now become the “final decision” meaning points rewarded by the PSS cannot be challenged (3) the high points awarded to headgear strikes usually results in most impacts taking place on the head (5 points). The work here aims to use the 20/20 Armor’s point scoring system, currently being used by over 1200 students in Canada and to create an algorithm for real-time scoring.

## 2. Materials and Methods

**Figure 1:**
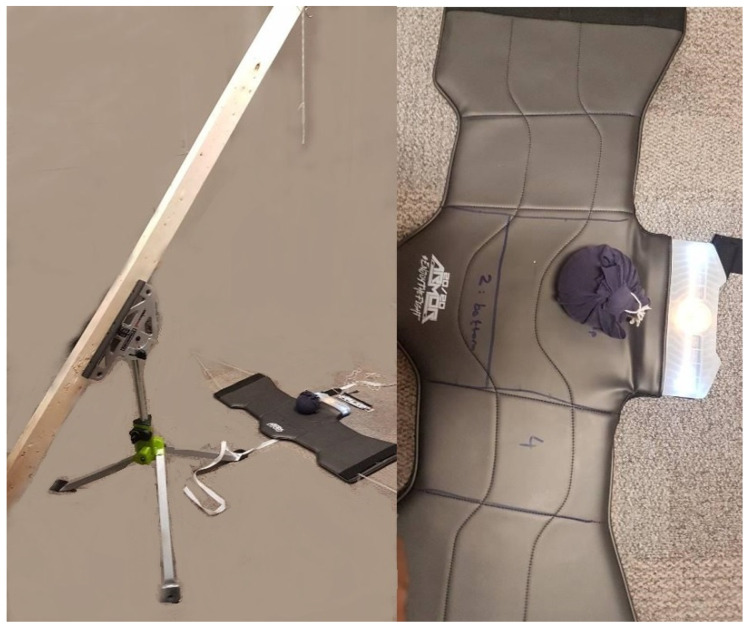
This figure shows the SARput drop-test apparatus (**left**) and the drop location (**right**). The vest is secured using anchor points.

A total of 3 studies were performed: Robustness Testing, Sensor Placement, and Classification of Impacts to the head. For the first study, martial arts standards (ISO 21924-3:2017) were used to design an apparatus called the SARput Apparatus, and is shown in Figure 1 [14]. For the second study, Sensor Placement, an electronic helmet (eHelmet), and an electronic glove (eGlove) were designed using the same IMU in different locations. For the last study, a data set was recorded of different impacts and then classified. It is important to note the first 2 studies were performed as a proof-of-concept for the final Classification of Impacts study. This is because as of March 2020, there are no impact data sets available for taekwondo requiring proofs-of-concept be performed. Athletes or the public were not involved in the design, conduct, reporting or dissemination plans of our research.

### 2.1. Robustness Testing

This study tested the robustness of the inertial measurement unit (IMU) being used in the helmet (LSM6DS3: STMicroelectrics). This is the same IMU used in the 20/20 Armor’s vest and hence the vest will be used for this proof-of-concept testing. Normally, the manufacturer of the IMU outlines the specifications (e.g., linear ranges) and are seen in Table 1. However, before any sensor can be used in research, its linear response and signal quality need to be validated especially considering the harsh environment faced in taekwondo. Our goal is to determine whether a linear relationship is observed when high energy impacts, such as those in taekwondo, are recreated. Basically, a weight is dropped from different heights to determine if higher impact energy generates a larger electrical signal. Following the ISO 21924-3:2017 standards, drop tests were chosen to isolate only the impact without noise from B.O.B. (Body Opponent Bag). If B.O.B. was used, the captured signal will comprise the initial impact plus the movement of B.O.B. after the impact. This means that we will capture unnecessary movement if a rigid body is not placed behind the sensor leading to false linearity testing. Therefore, it is important to have a rigid body (ground) behind the IMU to capture the deviations between different impact levels.

The data set includes 4 different heights (0.67 m, 1.13 m, 1.5 m, 1.75 m) resulting in 4 potential energy levels (29.5 J, 44.35 J, 59.3 J, 68.7 J). The 4 kg SARput was dropped from these heights onto a specific location on the vest. The vest was divided in 4 sections [1 (top), 2 (bottom), 3 (left) and 4 (right)] and the SARput was dropped onto Location 1 shown in Figure 1. A total of 101 signals were captured at 4 g and 250 dps. The collected accelerometer data are passed through a digital low-pass filter whose cut-off frequency is 742 Hz and the gyroscopic data are passed through a digital order 1 low-pass filter whose cutoff frequency is 60.2 Hz. The sampling frequency was set to these values and were determined by 1 of the 4 performance modes that the IMU can function at and the output data rate (sampling rate) to avoid aliasing was 1.66 kHz. It was important for our project to reduce the current consumption thus normal mode was selected consuming 900 µA. 

### 2.2. Sensor Placement

This study finalized the ideal location for IMU placement. Generally, the electronics can be placed in 2 possible areas, one on the body part used to strike the opponent (e.g., hand) and the other on the body part receiving the impact (head). In the former placement, the signal before the impact will be a major component to be considered in the classification algorithm which is representative of the strike technique and velocity. For the latter, the signal after the impact will constitute a major component for classification, which is the direct measurement of the force exerted on the opponent’s head/body. Another consideration is that the chosen location for the sensor needs to be outside the possible impact zone as hits directly to the electronics can destroy the equipment and/or injure the users. Furthermore, the design needs to be cost-effective as multiple sensors can be costly. Therefore, the purpose of this preliminary study is to determine the ideal location of sensor placement both qualitatively and quantitatively.

Century Fitness B.O.B. Body Opponent Bag—Body and Base—XL ($700) was selected as the dummy [17]. This dummy is commonly used in taekwondo schools as it replicates human properties well. The data set (B.O.B. Data Set 1) includes 20 impacts: 10 impacts with the IMU located on the eGlove, and 10 impacts with the IMU located on the eHelmet. In both locations, B.O.B. was used and the same jab punch was performed to the head. As the purpose of this study is to only determine which location results in usable signals, the hits performed at both sensor locations needs to be kept constant. A real-time data collection and classification algorithm were designed.

### 2.3. Classification of Impacts Using Second eHelmet Design

**Figure 2:**
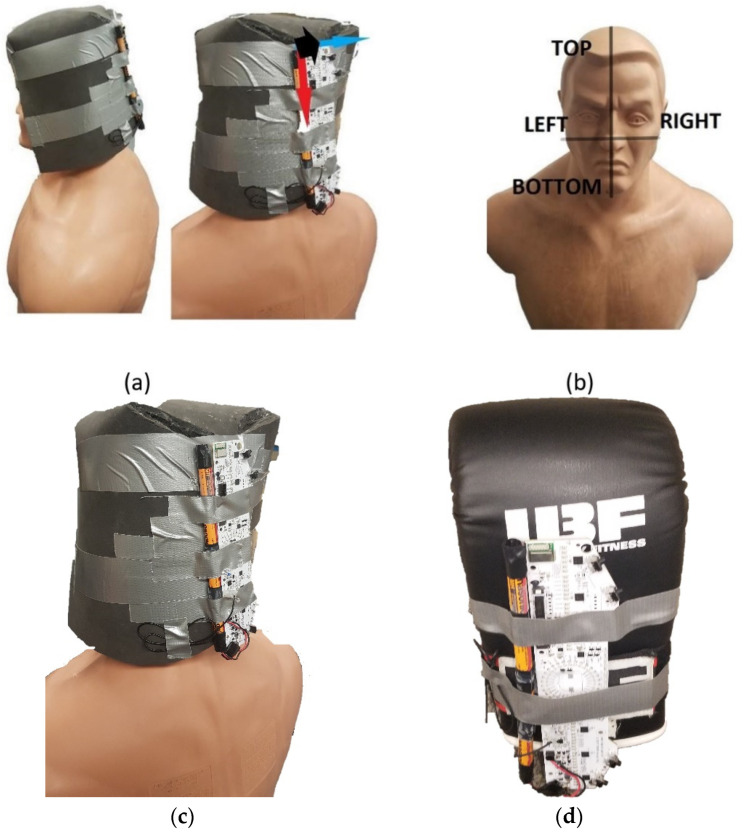
This figure (**a**) shows the eHelmet constructed from smart foam material as well as the x–y–z axis relative to the board. Note, red: *y*-axis, blue: *x*-axis, black: *z*-axis (out of page), (**b**) shows the impact locations on B.O.B, (**c**) displays a closeup of the helmet and (**d**) illustrates the board mounted to the glove secured in place with industrial level tape.

Once the ideal location was finalized from the previous study (Figure 2), work began on hit validation. Hit validation between legal vs. illegal hits presents a concern due to improper or accidental scoring, which is the foundation underlying the introduction of P.S.S. Therefore, the proposed research resolves the bias issue by creating a classification algorithm using an accelerometer and gyroscope.

A major task for our research involved data collection since no relevant data sets are available. In taekwondo, it is only legal to kick an opponent’s head which means any other type of hit to the head is illegal including punches. Hence, after consultation sessions with 2 Olympic taekwondo athletes who are instructors of the Master level, 2 vital classes were identified: Illegal (jab) vs. Legal (roundhouse kick). The orientation of the IMU was kept constant on the eHelmet. The IMU was then set to 8 g and 500 dps for improved signal quality based on Robustness Testing results. The features that were extracted included the following: Mean of the total acceleration (x, y, z) segmented for 150–300 N and 400–650 N where N represents samples, total number of minimum values for the gyroscope *z*-axis, energy concentration for the total acceleration, energy concentration for the total gyroscope (x, y, z) signal and energy concentration of gyroscope individual axis.

One constraint of the classification algorithm was that it should be a “lightweight” algorithm, which was set as a research objective by the SAR lab and 20/20 Armor team. Furthermore, the 20/20 Armor uses low-power and low-cost embedded systems where memory is at a premium. A lightweight algorithm refers to one that does not use machine learning or cloud computing. The algorithm was designed in-house employing a decision-tree approach. A decision tree consists of a root node, decision node, leaf node and branch. In the root node, the entire sample set is represented. In the decision, it becomes split into two nodes, and if there is no further split it is referred to as a leaf node. A branch is a subsection of the entire tree and is sometimes referred to as a sub-tree. It was based on the following extracted features: Mean of the total acceleration (x, y, z) segmented for 300 N and 400–650 N where N represents samples, total number of minimum values for the gyroscope *z*-axis, energy concentration for the total acceleration, energy concentration for the total gyroscope (x, y, z) signal and energy concentration of gyroscope individual axis. There is no need to store training data-sets on the flash memory. All computing is performed on the board as scoring needs to be instantaneous during matches. All punches and kicks were recorded in-house on B.O.B. For the development of the final classification algorithm, we use the full data sets collected in this study. The data set includes 50 kicks performed by a Master-level trainer and 50 punches to the head performed by a trainee (further information in Table 2a) under the supervision of the trainers (divided as shown in Table 2b). 

In total, 221 signals were captured to develop the classification algorithm. The statistical parameters used for classification evaluation include the confusion matrix, sensitivity, specificity, miss rate, fall out, positive predicted value and negative predicted value. Sensitivity (or recall or true positive rate) is defined as: True Positive (TP)/[True Positive (TP) + False Negative (FN)] while specificity (or true negative rate) is defined as: True Negative (TN)/[True Negative (TN) + False Positive (FP)]. False-positive rate (or fall out) is defined as 1-specificity. Miss Rate (or false-negative rate) is defined as: False Negative (FN)/[True Positive (TP) + False Negative (FN)]. Positive Predicted Value (or precision) is defined as: True Positive (TP)/[True Positive (TP) + False Positive (FP)]. Finally, Negative Predicted Value is defined as: True Negative (TN)/[False Negative (FP) + True Negative (TN)] [18]. Figure 2b shows the target areas on B.O.B.

## 3. Results

### 3.1. Robustness Testing

**Figure 3:**
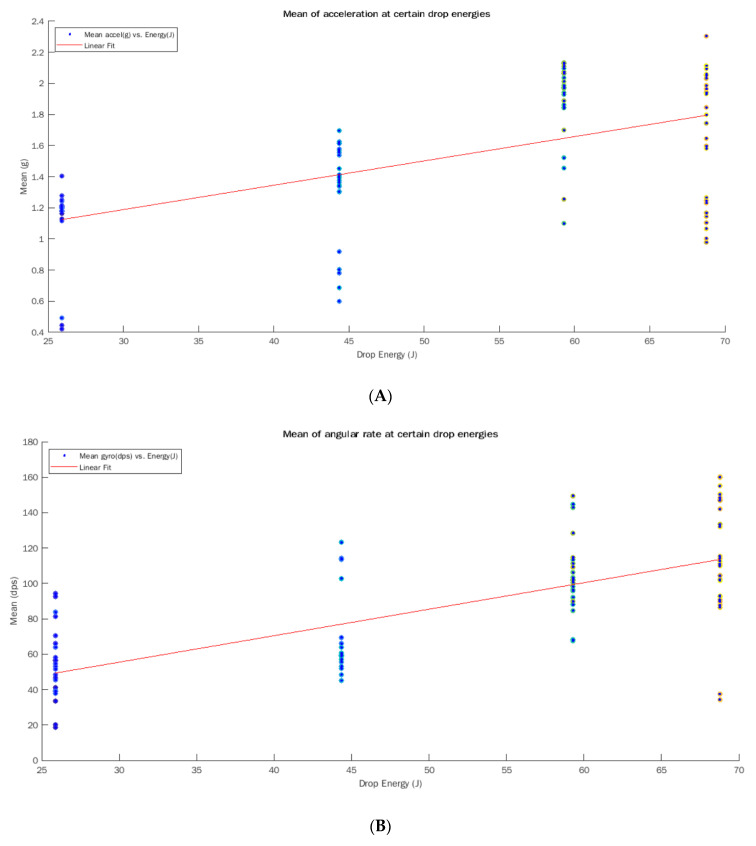
This figure shows all 101 signals with the linear curve for acceleration (**A**) and rotational rate (**B**) at 4 different heights of 0.67 m, 1.13 m, 1.5 m, and 1.75 m.

**Figure 4:**
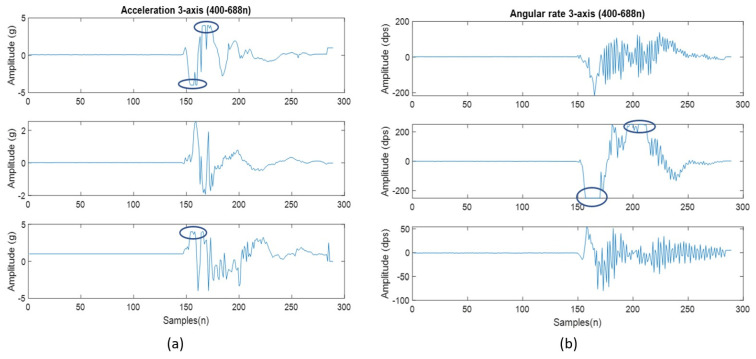
This figure shows the 3-axis and saturation points for (**a**) acceleration at 1.75 m and (**b**) gyroscope at 1.75 m.

The mean for each individual observation was calculated for both accelerometer and gyroscope signals. From there, the grand mean was calculated, and the analysis was performed on the data. The results are shown in Table 3 below. Once the means were calculated for all 101 drop-test signals, a linear model of degree 1 was estimated using the equation: *f*(*x*) = *mx* + *b* where *m* is 0.2554 and *b* is 1.497 for the acceleration data (Figure 3A). The same approach was chosen for the rotational data and the linear model was: *f*(*x*) = *mx* + *b* where *m* is 24.44 and *b* is 85.08 (Figure 3B). In addition to these results, Figure 4 shows the saturation points for the accelerometer (a) and the gyroscope (b). The circled areas demonstrate the saturation point (signal no. 2) from the drop height of 1.75 m for the settings at (4 g and 250 dps).

**Figure 5:**
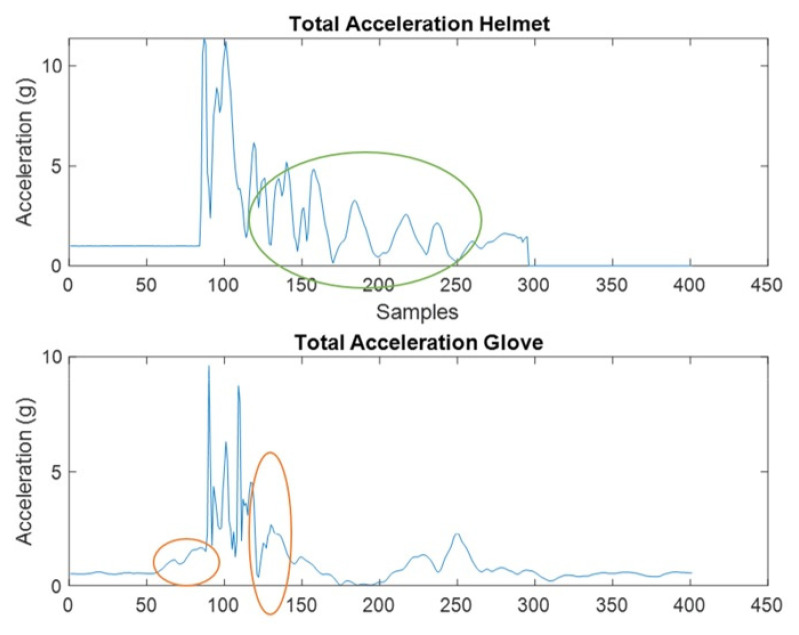
This figure shows the total acceleration of the helmet vs. the glove. Green circled area represents the cyclic motion of B.O.B as it oscillates after the punch while the orange circled areas represent the hand motion captured in the punches.

Figure 5 displays two example signals number 3 and number 13 (*x*-axis only). Signal 3 is from the IMU placed on the hand and signal 13 is from the IMU placed on the head while the jab is performed. The circled areas were of interest and an explanation is provided in the discussion.

Quantitative analysis was performed in the analyzing algorithm by the Hjorth Parameters as they bear good statistical properties used in the time domain and are also called normalized slope descriptors. Activity provides us with the variance (mean power) which represents a measure of the squared standard deviation of the amplitude. The next statistical parameter is mobility, which is the root mean square (RMS) of the slopes of the signal that is then divided by the root mean square of the amplitude. This can also be referred to as mean frequency and finally, the last parameter is complexity, which is the RMS of the rate of change of slope with reference to an ideal curve shape. This is possible since we know that the complexity of an ideal sine wave must be 1 and this results in an estimate of the bandwidth of the signal [19]. With the background in mind, the results are then shown in Table 4.

### 3.2. Classification of Impacts

**Figure 6:**
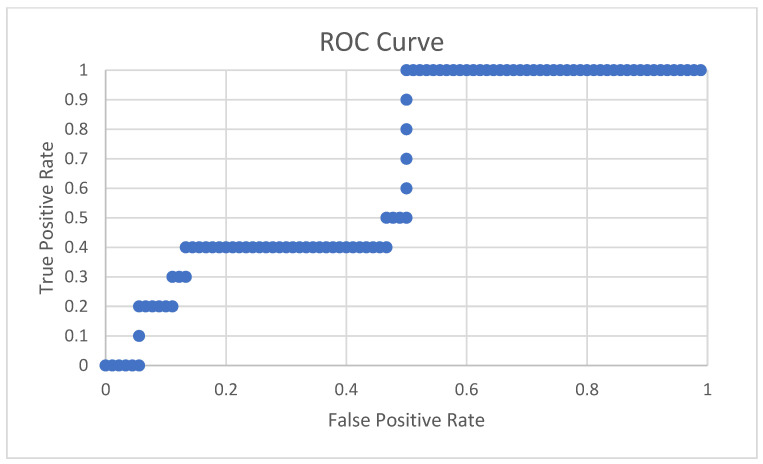
This figure shows the ROC curve for the classifier.

**Figure 7:**
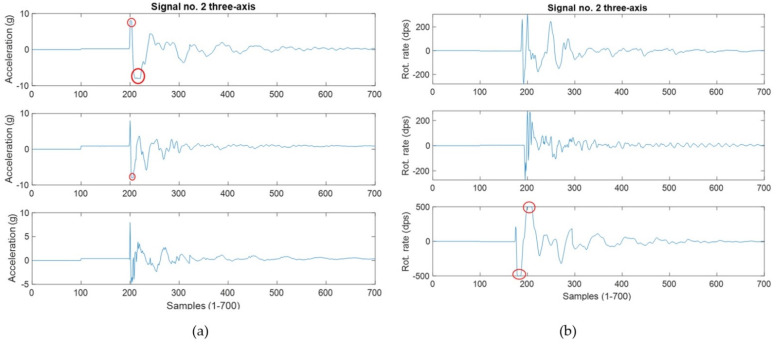
This figure shows the saturation point of 8 g for the accelerometer (**a**) on all 3-axes (X, Y, Z) circled in red and the gyroscope (**b**).

The designed algorithm was classified with an overall accuracy of 90%, misclassifying only 10 signals. The confusion matrix is shown in Table 5a and the sensitivity and specificity are calculated to be 90% each. Table 5b displays the rest of the statistical parameters. The ROC curve is shown in Figure 6 and the AUC (area under the curve) is 0.66 or 66% [20]. The saturation points are seen in Figure 7 that will be discussed in the following section. 

## 4. Discussion

### 4.1. Robustness Testing

The purpose of this initial study was to determine two factors: (1) can an accelerometer and gyroscope be used to detect impact level alone, and (2) is the relationship linear? We were able to observe that both the accelerometer and gyroscope can detect impacts in a linear fashion. There were a few key points observed: (1) a saturation point was noted in the accelerometer after dropping from a height of 1.51 m and above, (2) the gyroscope revealed a better linear response in our study when compared with the accelerometer. A saturation point was observed when the 4 kg shot put was dropped from 1.75 m. The signals for the accelerometer were clipped as the g-force exerted extends beyond the measuring capability of the sensor. The same was observed in the gyroscope however to a lesser extent. This saturation can be seen in Figure 4 by the circled areas. Observing the lower heights of 1.12 m and 1.33 m, shows they were linear for both the accelerometer and gyroscope, see Figure 3. The second question of whether this relationship linear, was also answered in this study. Considering the lower drop heights, the signals exhibit a linear gain with the increased impact level. This certainly proves the presence of a linear relationship, however, with a saturation.

### 4.2. Sensor Placement

It was concluded that the ideal location for sensor placement is the back of the head. Qualitatively employing industry experts, we were able to visually determine that the movement from the athlete’s hands and feet alone provide a limitation to the classification algorithm. This is because separating which movement contributed to the hit versus those which did not (i.e., the hit was dodged by the opponent) presents a difficult task requiring multiple sensors for validation and timing. This “noise” was high in our controlled jab punch and will only increase in real-world applications, as shown in Figure 5 (orange circled area). The motion of the hands and feet is significant in combat sports as they are used for movement, defense and striking the opponent. This is further supported by authors in [12] where they determined that differentiating complex movements such as grappling in combat supports represents a highly complex task, and there are no studies currently researching this [12]. From a durability point-of-view, placing the sensors on the hands/feet will result in high wear and tear. Next, this location requires 1 IMU as compared to 4 when placed on all hands and all feet. In terms of helmet sensor placement, in an effort to reduce the electronics in the impact zone, we chose a location that would be subject to the least impact, namely the back of the head.

When we quantitatively observe the results, we can determine that the mean activity (variance) is higher for the IMU placed on the head versus the IMU placed on the hand. This is in fact due to the oscillations seen by B.O.B. after an impact has occurred, as highlighted by the green circle. In real-world fights, the athlete will have a reaction to the impact, however, it will not be as excessive as B.O.B. and that will work in favor of our algorithm. The actual cost of the helmet with the IMU employed is roughly USD 11. Therefore, including that to the materials required to construct the helmet will prove extremely economical compared to the current point scoring system that costs roughly USD 2800–3400 each [11,13].

### 4.3. Classification of Impacts

The results provide a 90% accuracy of classification of different impacts using the accelerometer and gyroscope data in Version 1 of the eHelmet. The importance of this arises when we observe the scoring scheme of taekwondo and note that the highest points are awarded for kicks to the head. No complex machine learning was used, thus providing a sizable benefit. This allows our algorithm to be uploaded on the hardware allowing for (1) edge computing and (2) real-time classification. Edge computing refers to performing tasks on the microcontroller itself to avoid the need of transferring data to the cloud or remote servers for analysis. This bears several benefits such as security and processing time. Furthermore, this algorithm operates by using statistics-based features through a decision-tree approach that requires simple computations for classification.

In taekwondo, some impacts are known to reach 150 g or more; however, it was observed that the classification algorithm still performs well at 8 g and 500 dps [21]. An interesting observation was that even with these settings, there were still saturation points. This classification algorithm is not focused on the initial impact height, but rather the whole signal, thus significant information remained still available in the IMU signals. Only one or two axes usually reached the saturation point out of the 6. Increasing this range from 8 g to 16 g will certainly capture more data, but at the expense of decreasing the sensitivity of the IMU. Finally, it is advantageous to implement both an accelerometer and gyroscope in the helmet design. The head is supported by the neck, a pivot joint, and it faces significant rotational forces upon impact aiding classification. To further improve our classification for future studies, combining the NCF sensor data with the IMU will prove beneficial in classification as well as using a different IMU with a larger buffer to retain the data, which would allow for 16 g to be used with ease.

## 5. Conclusions

The three conclusions from this research are: (1) the IMU is capable of detecting taekwondo hits linearly up to a saturation point, (2) the ideal location for the IMU is the back of the head reducing costs and finally, (3) hit validation between legal and illegal hits was classified with 90% accuracy.

In addition, a significant advantage of our eHelmet is its facilitation of tele-coaching using the IoT concept. IoT refers to the Internet of Things and the ability to classify kicks directly on the vest will enable at-home coaching, where students can perfect their skills at home or a local gym and compare themselves based on similarity of their technique when compared with impacts performed by Olympic-level athletes. The inclusion of linearity testing in addition to hit validation in our work allows this to be possible. It is now possible to measure the kick impact level required for a valid hit, as well as proper technique since we can classify impacts. This improves upon the current approach of PSS that relies on a certain “threshold” required for legal impact. Our design is an all-in-one package using edge computing to classify impacts with work already beginning on the next generation.

## Figures and Tables

**Table 1:** Specifications for LSM6DS3.

Acceleration range (g)	±2–±16
Angular rate range (dps)	±125–±2000
Output data rate (kHz)	12.5–6.66
Power consumption	425 μA–1.25 mA
Buffer size (kbyte)	8
Temperature range (°C)	−40–+85
Dimensions (mm)	2.5 × 3 × 0.83
Linear Acceleration Sensitivity	±0.06–±0.488 mg/LSB
Linear Angular rate Sensitivity	4.375–70 mdps/LSB

References [15,16].

**Table 2:** **a.** Trainer details. **b.** Data set division.

**a**	
Master Level Trainer	
Age	43 y
Height	165 cm
Weight	150 kg
Level	Master
Experience	25+ y
Trainee	
Age	25 y
Height	187 cm
Weight	90 kg
**b**	
Punches	
Forehead	10
Right of head	15
Left of head	15
Face	10
Kicks	
Roundhouse kick	25
Back Kick (turning)	25

**Table 3:** Results Robustness Testing.

Drop Energies (Joules)	*29.5*	44.5	59.3	69.8
Accelerometer Grand Mean (G)	1.12	1.33	1.88	1.65
Gyroscope Grand Mean (dps)	54.35	65.96	105.05	113.81
R^2^ Accelerometer	0.35			
R^2^ Gyroscope	0.49			
Root Mean Square Error Accelerometer	0.35			
Root Mean Square Error Gyroscope	25.3			

**Table 4:** Hjorth parameters.

Helmet		Glove	
Accelerometer		Accelerometer	
Mean Activity	2.4456	Mean Activity	0.9354
Mobility	0.0940	Mobility	0.2565
Complexity	8.7101	Complexity	4.8710
Gyroscope		Gyroscope	
Mean Activity	11149	Mean Activity	1185
Mobility	0.0013	Mobility	0.0029
Complexity	18.5463	Complexity	21.5926

**Table 5:** **a.** Confusion Matrix for class 1 and class 2. **b.** Computed statistical parameters.

**a**		
	Class 1 (Legal)	Class 2 (Illegal)
Class 1	45 (TP)	5 (FP)
Class 2	5 (FN)	45 (TN)
	Sensitivity 90%	Specificity 90%
**b**		
Miss Rate	10%	
False positive Rate	10%	
Positive Predicted Value	90%	
Negative Predicted Value	90%	

## Data Availability

Data is available upon reasonable request.
